# Toward therapeutic electrophysiology: beta-band suppression as a biomarker in chronic local field potential recordings

**DOI:** 10.1038/s41531-022-00301-2

**Published:** 2022-04-19

**Authors:** Lucia K. Feldmann, Roxanne Lofredi, Wolf-Julian Neumann, Bassam Al-Fatly, Jan Roediger, Bahne H. Bahners, Petyo Nikolov, Timothy Denison, Assel Saryyeva, Joachim K. Krauss, Katharina Faust, Esther Florin, Alfons Schnitzler, Gerd-Helge Schneider, Andrea A. Kühn

**Affiliations:** 1 Department of Neurology, Charité—Universitätsmedizin Berlin, corporate member of Freie Universität Berlin and Humboldt-Universität zu Berlin, Berlin, Germany; 2 Berlin Institute of Health at Charité Universitätsmedizin Berlin, Berlin, Germany; 3 Charité—Universitätsmedizin Berlin, Einstein Center for Neurosciences Berlin, 10117 Berlin, Germany; 4 Institute of Clinical Neuroscience and Medical Psychology, Medical Faculty, Heinrich-Heine University Düsseldorf, Düsseldorf, Germany; 5 Movement Disorder and Neuromodulation Center, Department of Neurology, Medical Faculty, Düsseldorf, Germany; 6 MRC Brain Network Dynamics Unit, Nuffield Department of Clinical Neurosciences, University of Oxford, Oxford, United Kingdom; 7 Department of Neurosurgery, Medical School Hannover, Hannover, Germany; 8 Department of Neurosurgery, Charité—Universitätsmedizin Berlin, corporate member of Freie Universität Berlin and Humboldt-Universität zu Berlin, Berlin, Germany; 9 Berlin School of Mind and Brain, Charité Universitätsmedizin Medicine, Berlin, Germany; 10 NeuroCure Clinical Research Centre, Charité Universitätsmedizin, Berlin, Germany; 11 DZNE, German Center for Degenerative Diseases, Berlin, Germany

**Keywords:** Parkinson's disease, Neuroscience

## Abstract

Adaptive deep brain stimulation (aDBS) is a promising concept for feedback-based neurostimulation, with the potential of clinical implementation with the sensing-enabled Percept neurostimulator. We aim to characterize chronic electrophysiological activity during stimulation and to validate beta-band activity as a biomarker for bradykinesia. Subthalamic activity was recorded during stepwise stimulation amplitude increase OFF medication in 10 Parkinson’s patients during rest and finger tapping. Offline analysis of wavelet-transformed beta-band activity and assessment of inter-variable relationships in linear mixed effects models were implemented. There was a stepwise suppression of low-beta activity with increasing stimulation intensity (*p* = 0.002). Low-beta power was negatively correlated with movement speed and predictive for velocity improvements (*p* < 0.001), stimulation amplitude for beta suppression (*p* < 0.001). Here, we characterize beta-band modulation as a chronic biomarker for motor performance. Our investigations support the use of electrophysiology in therapy optimization, providing evidence for the use of biomarker analysis for clinical aDBS.

## Introduction

Deep brain stimulation (DBS) to the subthalamic nucleus (STN) provides an effective therapy for patients with advanced Parkinson’s disease (PD), suffering from symptom fluctuations and dopaminergic side effects^1^.

Previously, externalization of DBS leads in a brief post-operative interval has allowed the characterization of disease-specific exaggerated oscillatory activity in PD in the beta frequency band (13–30 Hz). It could be shown that beta-band activity is modulated through therapy—both stimulation and medication^2–5^—and that these changes are correlated with improvement in motor symptoms, particularly bradykinesia^6–9^. The long-term evaluations of beta frequency band activity conducted so far have shown similar dynamics; most chronic recordings have been performed using the Medtronic Activa PC + S/RC + S system^10–14^ or PINS Medical devices^15^, additional chronic recordings with temporarily externalized patients were conducted for up to 24 h with the Newronika AlphaDBS system^16–18^.

Beside technical developments, e.g. regarding electrode design, the increased knowledge of electrophysiological characteristics has inspired the development of feedback stimulation algorithms beyond conventional DBS (cDBS)^19^. Adaptive DBS (aDBS) relies on the online analysis of electrophysiological biomarkers as feedback signals for neurostimulation. As beta frequency band activity is modulated through therapy and correlates with motor improvement, it may be a viable biomarker. Recent studies have investigated different closed-loop algorithms, demonstrating the superiority of aDBS over cDBS^18,20^, reducing side effects^21–23^ and energy delivered to the tissue^24^. Most studies were performed post-operatively in short-term laboratory settings^16,20–22^ at a maximum of 8 h of aDBS^16^. One study used an implantable pulse generator (IPG)^24^ and one was conducted in patients at the time of surgical IPG replacement^23,25^.

After decades of neurophysiological research, technical development can now translate scientific findings into therapy: The new Percept^TM^ IPG (Medtronic, MN, USA) is a sensing-enabled neurostimulator, which will allow long-term therapy with aDBS in chronically implanted patients for the first time. Currently, chronic sensing is available and the easy access to electrophysiological recordings on any clinician programmer tablet may broaden the use of intracranial electrophysiology as a clinical diagnostic tool for objective monitoring of symptom severity and accelerate DBS parameter optimization.

In this study, we characterize beta frequency band activity as a biomarker in chronic recordings using the Percept IPG. So far, there is limited knowledge on the relation between stimulation amplitudes, beta-band activity and motor performance in PD patients with chronic DBS. However, while the first clinical study investigating chronic aDBS is under way^26^, this is an essential prerequisite for aDBS with stimulation amplitude modulation based on beta-band signals. Here, we investigate the characteristics of beta-band modulation during stepwise increase of stimulation amplitude in 10 PD patients implanted with the Percept IPG.

## Results

### Stimulation exclusively suppresses beta frequency band activity in a dose-dependent manner

**Fig. 1:**
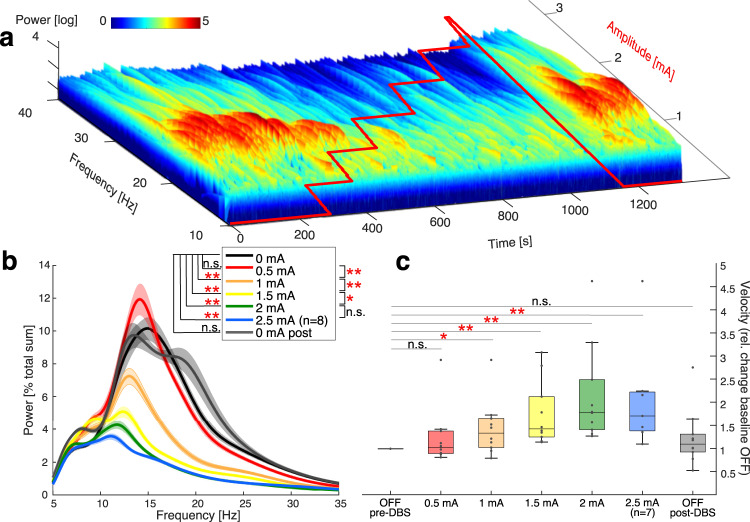
Stepwise stimulation increase is mirrored in the stepwise suppression of beta frequency band activity and stepwise improvement of motor performance. **a** Stimulation amplitude layered as a red line on the time–frequency plot of a representative recording session in a PD patient during monopolar review **b** Averaged power spectra for 30 sec mean resting state power per stimulation step across bradykinetic patients (10 STN), (mean beta-band activity [% total sum] by stimulation step: OFF: 4.31 ± 1.2%, 0.5 mA: 4.09 ± 1.06%, 1 mA: 2.27 ± 0.83%, 1.5 mA: 1.53 ± 0.54%, 2 mA: 1.17 ± 0.41%, 2.5 mA: 1.12 ± 0.41%, OFF post-DBS: 4.64 ± 2.22%) **c:** Mean velocity improvement in bradykinetic patients (mean velocitiy relative to baseline 0.5 mA = 1.24±0.63; 1 mA = 1.44 ± 0.6; 1.5 mA=1.76 ± 0.69; 2 mA = 2.15 ± 1.06; 2.5 mA = 2.1 ± 1.2, OFF post-DBS: 1.24 ± 0.61, central mark in the boxplot is the median, edges are 25th/75th percentile), with paired permutation testing.

**Fig. 2:**
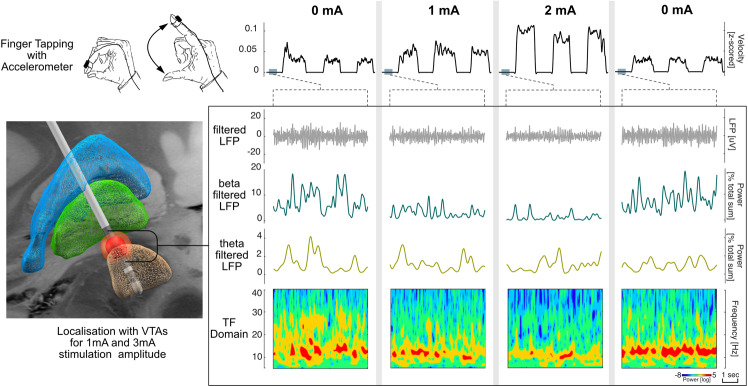
Beta frequency band suppression during stimulation corresponds to bradykinesia improvement. Representative example of analyzed data per stimulation level. Columns represent steps of increasing stimulation amplitude: stimOFF–1 mA stimON–2 mA stimON–stimOFF. Upper trace shows smoothed velocity traces of finger tapping. Resting state LFP-recordings from contact pair 1–3 are shown as (i) filtered LFP (5–48/52–98 Hz), (ii) beta filtered LFP (13–35 Hz), (iii) theta band filtered LFP (5–8 Hz) as control frequency, and iv) time-frequency representation; left: DBS localization and VTA reconstruction for 1 and 2 mA, visualized as previously described^57^.

We observed a successive suppression of beta-band activity with stepwise increase in stimulation amplitude in each patient. A representative example time–frequency plot is shown in Fig. 1a. When averaged across patients, mean beta-band activity decreased with each step of increasing stimulation amplitude (Fig. 1b). Group sizes decreased towards higher stimulation amplitudes as recordings were only performed up to the side effect threshold. While 0.5 mA stimulation did not significantly decrease mean beta frequency band activity (OFF/0.5 mA: *p* = 0.49), mean beta-band activity was significantly suppressed with 1.0 mA stimulation amplitude, with a further stepwise decrease at 1.5 mA and 2 mA (OFF/1 mA: *p* = 0.008; OFF/1.5 mA: *p* = 0.002; OFF/2 mA: *p* = 0.002) in parallel with an increasing clinical effect on motor performance in the bradykinetic patients (Fig. 1c). A significant motor improvement was observed, with the best clinical effect at 1.5 mA and 2 mA stimulation intensity (OFF/1 mA: *p* = 0.03; OFF/1.5 mA: *p* = 0.002; OFF/2 mA: *p* = 0.002, OFF/2.5 mA: *p* = 0.016). After cessation of stimulation, beta-band activity returned to baseline levels (OFF pre-DBS/OFF post-DBS: *p* = 0.97), which was reached after 13 s in 60% of patients. In an individual patient, Fig. 2 illustrates how the resting state electrophysiological data corresponds with increased velocity in the finger-tapping task recorded with an accelerometer.

**Fig. 3:**
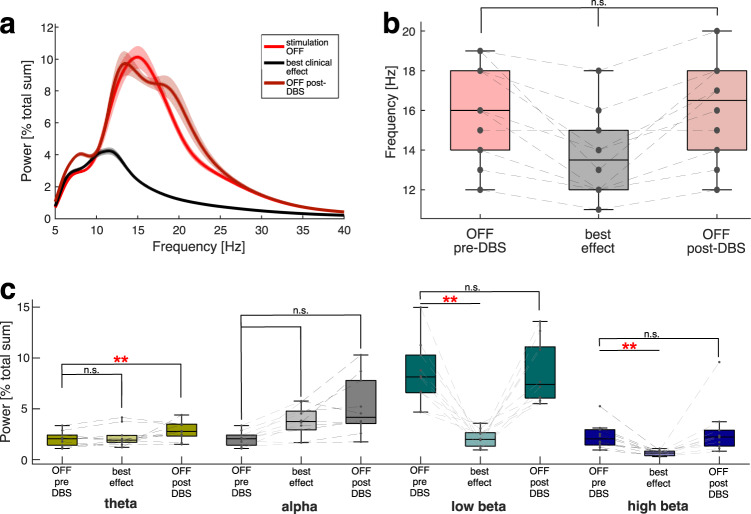
Chronic DBS suppresses beta frequency band activity. **a** Mean power spectra are grouped according to clinical effect of stimulation as measured by finger tapping showing that effective stimulation suppresses beta-band activity. **b** Peak beta-band amplitude is suppressed during effective DBS and spectral peaks are shifted towards lower frequencies (mean peak frequency OFF: 16 ± 2.5 Hz; mean peak frequency ON: 13.7 ± 2.16 Hz, mean peak frequency post-DBS: 16 Hz ± 2.5 Hz), with no significant differences). **c** Stimulation modulates mean power spectra in a frequency-specific manner with significant suppression of low and high-beta-band power but not alpha or theta band activity. Boxplots with median as the central mark, 25th/75th percentile as edges. Low-beta-band (13–20 Hz) and high-beta-band activity (20–35 Hz) was significantly suppressed through DBS at the stimulation intensity for best clinical effect (low-beta OFF-DBS: 8.43±3.14 [% total sum]/best clinical effect: 2.11 ± 0.85 [% total sum]; *p* = 0.002; mean high-beta OFF-DBS: 2.32 ± 1.3 [% total sum], mean high-beta best clinical effect: 0.66 ± 0.25 [% total sum], *p* = 0.002), no significant modulation in the theta (5–8 Hz) and alpha (8–12 Hz) band activity (OFF/best clinical effect, mean theta OFF-DBS: 2.04 ± 0.71 Power [% total sum], mean theta best clinical effect: 2.24 ± 0.96 Power [% total sum]; *p* = 0.26, mean alpha OFF-DBS: 4.65 ± 3.6 Power [% total sum], mean alpha best clinical effect: 3.79 ± 1.43 Power [% total sum]; *p* = 0.475).

Since the clinical effect occurred individually at different stimulation amplitudes, we averaged the beta-band activity with respect to the clinical effect of stimulation based on the velocity analysis from accelerometer recordings. Best clinical effect was defined, power spectra for 10 hemispheres with motor improvement were averaged (Fig. 3a). As differential modulation for low-beta and high-beta-band activity have been suggested previously, we investigated both frequency bands separately. Low-beta-band (13–20 Hz) activity was significantly suppressed through DBS at the stimulation intensity for best clinical effect (low-beta OFF-DBS: 8.43 ± 3.14 [% total sum], low-beta/best clinical effect: 2.11 ± 0.85 [% total sum]; *p* = 0.002). Similarly, high beta (20–35 Hz) was significantly modulated through DBS (mean high-beta OFF-DBS: 2.32 ± 1.3 [% total sum], mean high-beta best clinical effect: 0.66 ± 0.25 [% total sum]; OFF/best clinical effect *p* = 0.002) (Fig. 3c). This effect was frequency-specific to the beta band, with no significant modulation through DBS observed in the theta (5–8 Hz) and alpha (8–12 Hz) band activity (OFF/best clinical effect, mean theta OFF-DBS: 2.04 ± 0.71 Power [% total sum], mean theta best clinical effect: 2.24±0.96 Power [% total sum]; *p* = 0.26, mean alpha OFF-DBS: 4.65 ± 3.6 Power [% total sum], mean alpha best clinical effect: 3.79 ± 1.43 Power [% total sum]; *p* = 0.475). There was a significant difference between theta band activity before and after DBS (*p* = 0.002) which could be considered a rebound in post-DBS low-frequency activity as previously observed, albeit non-significant^3^. Similar results were obtained when removing outliers in the theta (*n* = 2) and high-beta range (*n* = 1).

While all patients presented with a peak in the beta frequency range, there was a variation in the individual peak frequencies (Fig. 3b) (mean peak frequency OFF: 16 ± 2.5 Hz (range: 12–19 Hz)). During DBS at the best clinical effect level, the peak frequency shifted towards lower frequencies (mean peak frequency ON: 13.7 ± 2.16 Hz (range: 11–17 Hz)), this difference in the peak frequency was however not significant (Peak OFF-pre-DBS/ Peak ON DBS *p* = 0.083; Peak ON DBS/OFF-post-DBS *p* = 0.083). Post-DBS, the mean peak returned to the initial frequencies 16 Hz ± 2.5 Hz (Peak OFF-pre/Peak OFF-post non-significant, *p* = 1.2).

### Beta-band suppression is a predictor for improved bradykinesia

**Fig. 4:**
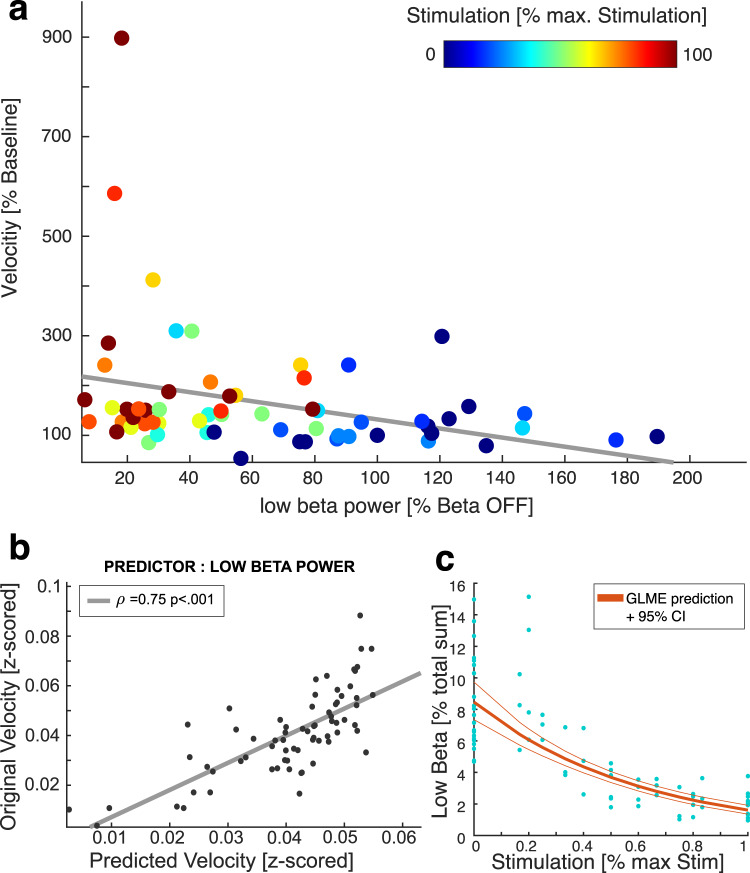
Suppression of low-beta activity corresponds with motor improvement. **a** Scatter plot summarizing interrelation between motor performance, beta suppression and stimulation intensity for the patient cohort, stimulation is presented in % of maximal stimulation, which was the maximum tolerable intensity. **b** Linear mixed effects model shows that low-beta-band activity is a strong predictor for velocity improvement, while the relationship between stimulation intensity and beta suppression follows a logarithmic relation (**c**).

In the group of patients with bradykinetic symptoms, Fig. 4a illustrates the correlation of stimulation-induced beta power suppression with improved motor performance and its dependence on stimulation amplitude, i.e. beta activity suppression is associated with stepwise motor improvement in parallel with increasing stimulation amplitudes. In order to define the predictive value of beta activity for motor performance, we calculated a linear mixed effects (LME) model. Across patients, low-beta power significantly explained ~50% of the variance in movement velocity in our LME model with random intercept (Coefficient Estimate: −0.002, *R*² = 0.49, *P* < 0.001, Fig. 4b). While high-beta power was a significant predictor as well (Coefficient Estimate: −0.004, *P* = 0.0013), a direct model comparison suggested a slight superiority of low-beta power as predictor for movement velocity (LME with low-beta power: BIC −393.35, LME with high-beta power: BIC −380.57, ∆BIC > 6 = strong evidence for superiority). The LME model also revealed alpha to be predictive for bradykinesia, (Coefficient Estimate: −0.002, *P* = 0.002, BIC = −380.53), while theta was not predictive (Coefficient Estimate: −0.002, *P* = 0.51, BIC = −371.18). After removal of one STN with a large 12 Hz peak (see Table 1), the model was not significant for alpha-band activity (Coefficient Estimate: −0.002, *P* = 0.9, BIC = −19.35).

**Table 1:** Demographic and clinical subject information.

Patient ID	Age (y)	Sex	PD type	DD (y)	LEDD (mg)	UPDRS-III OFF/ON pre-DBS	UPDRS-III^a^ MED OFF/ STIM OFF	UPDRS-III^b^ MED OFF /STIM ON	Stimulation contact R/L	Beta peak (Hz)	Exclusion/inclusion
1	61	f	akinetic-rigid	10	775	50/9	22	15	1	–	ECG/Stimulation Aliasing
									10	19	YES
2	67	m	akinetic-rigid	11	1250	63/30	48	34	2	16	YES
									10	15	YES
3	56	f	akinetic-rigid	15	150	37/23	29	20	1	16	YES
									9	–	ECG/Stimulation Aliasing
4	63	f	akinetic-rigid	7	525	51/20	42	24	2	18	YES
									10	18	YES
5	57	f	akinetic-rigid	13	300	67/28	78	47	1	14,27	YES
									9	–	Patient tiredness
6	70	m	tremor-dominant	16	150	40/31	63	39	2	–	YES
									9	–	ECG/Stimulation Aliasing
7	49	f	tremor-dominant	6	150	43/28	63	35	2	–	YES
									9	–	YES
8	61	f	akinetic-rigid	6	300	50/33	40	19	2	19	YES
									9	–	ECG/Stimulation Aliasing
9	65	m	akinetic-rigid	15	300	66/27	39	14	2	13,24	YES
									10	12,24	YES
10	53	m	tremor-dominant	7	500	42/21	48	30	1	–	Stimulation Aliasing
									10	–	Stimulation Aliasing

^a^MDS-UPDRS Part III Scores at the time of recording represented as Medication/Stimulation, stimulation OFF at least 30 min OFF DBS.

*DD* Duration since diagnosis, *LEDD* levodopa equivalent daily dose, *DBS* deep brain stimulation, visually detected beta peaks are presented for the 10 STN included in the main analysis.

Finally, we reconfirmed the interrelation between stimulation amplitude and low-beta suppression using a generalized LME. When fitted to a linear model, stimulation amplitude was a strong predictor for low-beta reduction (Coefficient Estimate: −1.66, *R*² = 0.71, *P* = < 0.0001) (Fig. 4c). Stimulation amplitude was also a predictor for high-beta-band reduction (Coefficient Estimate: −1.41, *R*² = 0.71, *P* = 4.4^−16^), while theta band modulation, as a control frequency, was not predictive (*P* = 0.32), alpha being significant (*P* = 0.02, after outlier removal *P* = 0.048). There was a superiority upon model comparison regarding high beta (low-beta BIC: 82.14, high-beta BIC 103.35, ∆BIC > 6).

## Discussion

In this study, we assessed changes of beta-band activity as a biomarker for bradykinesia in PD patients using chronic sensing with the Percept IPG. The Percept bears the potential of aDBS and could be used for electrophysiological therapy monitoring. This study supports this new technology and lays the foundation for the clinical use of chronic biomarker sensing for aDBS in bradykinetic patients. Importantly, we showed a tight relation of dose-dependent stimulation-induced beta-band suppression to stepwise motor improvement as a major prerequisite for aDBS algorithms. This titrated DBS effect on beta-band activity and its relation to the clinical outcome is in line with previous investigations of beta frequency band activity as a potential biomarker in peri-operative settings^2,7,27,28^ and in chronically implanted patients^10–12,29,30^. While Eusebio et al. used a similar recording protocol, they investigated beta suppression acutely peri-operatively and without a motor task^28^. Further research in chronically implanted patients focused on different motor states^30^, gait and changed stimulation frequencies at constant stimulation amplitude^29^, or beta dynamics during movement^10,12^. So far, this has not been shown using chronic beta-band rest recordings in an IPG suitable for clinical aDBS. Thus, our findings support beta-band activity as a valuable biomarker for aDBS and contribute substantially to the translation of electrophysiology into clinical practice towards optimized personalized DBS therapy and ultimately, the chronic implementation of aDBS.

In our study, we used an accelerometer for objective measures of bradykinesia during a finger-tapping task. During the stepwise increase of stimulation, a dose-dependent improvement in motor behavior occurred in all patients and was related to the frequency-specific suppression in STN beta-band activity. In line with other studies^3^, theta and alpha-band activity was not significantly modulated by DBS.

Interestingly, beta-band suppression was significant for the whole beta band (13–35 Hz) but more pronounced in the low-beta band (13–20 Hz) as compared to the high-beta band (20–35 Hz) which is similar to a previous observation of chronic levodopa-induced beta-band suppression^31^. Importantly, low-beta-band suppression was also the strongest predictor explaining ~50% of the variance in movement velocity in our LME model, superior to high-beta-band activity. Interestingly, in the LME model, alpha-band activity was also a significant predictor for motor outcome, which may be due to an overall high percentage of low-beta-band peaks in the included patients (see Table 1), the activity of which would spread to the alpha band. After removal of the STN with the peak at the border between alpha and beta band (12 Hz) the model was no longer significant for alpha activity as a predictor of motor outcome but results were consistent for the beta range. There have previously been heterogeneous results in the literature, with some studies including 8–12 Hz into the broad sub-beta/beta band^8,32,33^, that correlated with motor performance. As demonstrated in Fig. 3c, there is no consistent suppression of low-frequency activity across STNs. Overall, beta-band activity seems to be a more stable biomarker for chronic sensing enabled devices such as Percept that use a 5 Hz bin frequency band for the feedback signal^34^.

Increasing stimulation intensities suppress beta-band activity during movement^10^. As suggested by our results, also resting state beta-band activity allows a distinct monitoring of motor performance and titration of stimulation intensity. As resting state assessments would be advantagous in practical implementation, resting state peak beta-band activity could also serve as an easily accessible biomarker for DBS parameter optimization and should also be reliable for aDBS calibration.

We observed a shift of peak frequency activity towards lower frequencies at a clinically effective stimulation amplitude although this effect was not significant. Similarly, it has been suggested that low-beta-band frequency modulation, additionally to amplitude modulation, may be crucially involved in movement-related changes in basal ganglia network dynamics^35^. This effect of frequency was further modulated by levodopa^31^. Similar dynamics during DBS have been controversially discussed^3,32,36,37^. Importantly, in our patients the beta peak shifted back to pre-stimulation frequencies after cessation of DBS. This would also suggest that the selection of the OFF-medication/OFF stimulation peak frequency for chronic monitoring in aDBS, as implemented in the Percept, would suffice as a biomarker for therapy control. However, the frequency effect should be evaluated in larger studies over longer time periods since a stable beta peak is an important prerequisite for chronic sensing in aDBS.

A concern when sensing physiological signals in the presence of stimulation is channel saturation. If the sensing channel is corrupted by excess stimulation artifact, it can potentially lead to distortion or false-triggering of the adaptive algorithm^38^. Artifact generation is complex interplay of stimulation amplitude, absolute impedance levels, and relative matching between the measurement electrodes. In this study, the maximum current delivered was 2.5–3 mA unipolar to the case. This stimulation is approximately 50% below the level that creates signal chain effects with measurement electrode pairs with worst-case mismatch^39^. In the presence of excess stimulation, the properties of the signal chain lead to an increase in the measurement noise floor in the presence of excess artifact as illustrated in the characterization data in Supplementary Figure 1. This elevation of the measurement floor was not observed in the cases from this study. Even when the noise is increased, the signal amplitudes and frequency characteristics are not impacted until significantly greater stimulation levels, greater than 10 mA, are applied, which provides confidence in the validity of the biophysical interpretation of the data.

One limitation of this study is that we excusively focused on bradykinesia for motor performance assessment during titration of stimulation. Other motor features such as tremor or freezing of gait may be reflected by different biomarkers^23,40–42^. Local field potential (LFP)-signatures in tremor-dominant PD patients have been more variable with beta band but also gamma and theta band activity related to the tremor activity^23,41–43^. Furthermore, it should be noted that, while all recordings for our main analysis were performed during a three months follow-up routine visit, two tremor-dominant patients were included after IPG replacement, years after DBS electrode implantation (see Supplementary Figure 2). Similar to previous studies suggesting stability of LFP beta activity in chronic recordings^32,44,45^, these patients showed a distinct beta peak. STN LFP in the two tremor-dominant patients showed a consistent pattern of beta-band modulation by DBS but inconsistent effects on bradykinesia as measured with the accelerometer that was contaminated by tremor activity. For this reason, both patients were excluded from the main group analysis (respective power spectra demonstrating suppression in the beta frequency band can be found in Supplementary Fig. 2)

Here, we only investigate beta band changes during the OFF-medication state at rest. Beta-band activity is already strongly suppressed through dopaminergic medication^8,31,36^ with the need for reduced stimulation amplitude to reach motor improvement^46^ and avoid dyskinesia^16,21^. Effects of movement and medication on beta-band activity as well as biomarkers in other frequency bands, e.g. the gamma band^47^ in the medication ON state and during different motor activities need to be investigated for future clinical application of aDBS.

Data quality was compromised by electrocardiogram (ECG) artifacts in 4 subthalamic nuclei of 4 patients, three of which were implanted with the IPG on the left side. Recent technical investigations into likelihood of ECG contaminations^48^ showed the predominance of ECG artifacts with left-sided implants and laterality of IPG placement should be taken into consideration in future for patients with chronic sensing devices. However, although previous aDBS studies have suggested independent oscillatory activity between hemispheres^49^, other studies have demonstrated beta coupling between subthalamic nuclei^5,50^ and even suppression of oscillatory activity in the contralateral STN during unilateral DBS^51^. Hence, even unilateral robust DBS signals might be a sufficient basis for aDBS algorithms.

In this study, we provide evidence for beta-band activity as a viable biomarker for motor improvement in bradykinetic-rigid PD patients using the Percept IPG. We show that beta frequency band activity is specifically suppressed through effective DBS, and that beta frequency band suppression is a strong predictor for bradykinesia improvement. Hence, this study paves the way to the chronic implementation of aDBS in bradykinetic-rigid PD patients using the Percept IPG. Furthermore, we illustrate changes in biomarker activity in relation to motor improvement and stimulation, which might have direct implications for electrophysiology-guided clinical DBS parameter optimization.

## Methods

### Subjects

All subjects gave written informed consent and the study was approved by the local ethics committees of the Charité Universitätsmedizin Berlin (EA2/256/60) and the Medical Faculty of Heinrich-Heine-University Düsseldorf (Study No. 2019-629_2), and was conducted in accordance with the ethical standards set by the Declaration of Helsinki.

10 PD patients (6 females/4 males) from 3 DBS centers were included in the study. All patients underwent implantation of bilateral DBS electrodes in the STN^52^ and patients were implanted with Medtronic 3389 DBS leads, which were connected with the sensing-enabled Percept IPG (Medtronic, Minneapolis, MN, USA). Contact 0 in the right and 8 in the left hemisphere were the lowermost contacts, 3 and 11 the uppermost contacts, respectively.

The mean age of the participants was 60.2 ± 6.2 years (mean ± SD) with a mean disease duration of 10.6 ± 3.8 years (see Table 1 for further details). Previous studies confirmed stability of beta-band activity for at least 5 years^34,45^ and correlation between disease duration and beta-band suppression at stimulation intensities for the best clinical effect was not significant. The mean pre-operative OFF medication UPDRS-III score was 50.9 ± 10.4. During the recordings at a chronic post-surgical timepoint of at least 3 months, MDS-UPDRS - III was assessed OFF medication with STN DBS ON (UPDRS-ON = 27.7 ± 10.5) and OFF (UPRS-OFF = 47.2 ± 16).

Recordings were performed at the 3-month follow-up in 8 patients (Subject 1–5, 8–9), and during outpatient clinic visits in Subject 6 and 7 who were implanted with the Percept IPG at a regular IPG exchange (DBS duration at IPG exchange 5 and 10 years). The placement of DBS leads within the STN was determined by intraoperative microelectrode recordings and was confirmed by post-operative imaging with fusion of pre-operative MRI and post-operative CT in all patients (see Supplementary Fig. 1) using Lead-DBS-v2^53^. To do so, all postoperative CT images were first linearly coregistered to their corresponding preoperative MRIs using Advanced Normalization Tools^54^ and manually refined when necessary. Pre- and postoperative images were then normalized into ICBM 2009b NLIN asymmetric space using the symmetric diffeomorphic image registration approach in ANTs implemented in Lead-DBS. Electrode localization within the STN and active contacts during the monopolar review recordings are visualized in Supplementary Fig. 3.

### Experimental protocol and data acquisition

All recordings were performed separately for each hemisphere after withdrawal of dopaminergic medication for at least 12 h (OFF medication state). Recordings OFF-stimulation were performed after a washout-phase of at least 30 min, which was reported to be sufficient for recovery of at least 75% of motor symptoms^55^ and a stabilization of beta-band activity^3,56^. In subject 5, the task was only performed with stimulation of the right hemisphere (due to fatigue).

Initially, we evaluated the artifact status and the power spectra generated for all possible recording configurations in the BrainSense Survey/BrainSense Signal Check modes using the Medtronic clinician programmer. During chronic sensing only the middle two contacts can be used for stimulation. From the contact pairs adjacent to the two possible stimulation contacts (right: 1 + 2/left: 9 + 10), we selected the bipolar contact pair with the highest beta peak for the recordings (right: 0–2, 1–3; left: 8–10, 9–11). Stimulation contacts used in this study are presented in Table 1.

During the monopolar review, patients were seated comfortably in an arm chair. Following a rest recording of 60 s, patients conducted 2–3 blocks of 10 s finger tapping (MDS Unified Parkinson’s Disease Rating Scale (UPDRS)-III item 3.7/3.8) of both hands, with 10 s rest between each block. For each hemisphere in separate recordings, the stimulation was unilaterally increased in steps of 0.5 mA (Fig. 1) up to the presentation of side effects. On each stimulation level, the rest and finger-tapping assessments, as described above, were conducted. To avoid switch artifacts, stimulation was ramped down to off stimulation (average time 23 ± 6.64 s).

LFPs were recorded using the Percept IPG, data were sampled at 250 Hz, streamed to the Medtronic clinician programmer, exported to the json-file format and saved to a personal computer. The motor task performance was objectified using 3-D accelerometers (n = 7: TMSi, The Netherlands; n = 1: Alpha Omega, Nazareth, Israel; n = 2: Analog Devices, Norwood, USA). Additionally, the improvement of overall motor performance during stimulation was assessed using MDS UDPRS Part-III scores OFF medication.

### Data analysis and statistical analysis

All data were analyzed offline in MATLAB (The Mathworks, Nattick, Massachussets). Electrophysiological data were analyzed using the open-source Perceive toolbox (https://github.com/neuromodulation/perceive/) and the statistical parametric mapping toolbox (SPM12, UCL, London, UK). Data were inspected visually and recordings with ECG contamination were identified in relation to the delta oscillatory activity and beta-band activity, subjects with strong stimulation aliasing were excluded (see Table 1). From 19 hemispheres of 10 PD patients, recordings from 6 hemispheres from 5 subjects were excluded due to artifact contamination with strong stimulation aliasing, which is in line with previously reported artifact contamination levels in recordings with IPGs^48^. For a homogenous patient population, 10 STN from the bradykinetic patients are included in the final analysis. A 5^th^ order Butterworth filter was applied at 5 Hz highpass and 98 Hz lowpass to avoid movement artifacts and stimulation aliasing, a bandstop filter was applied at 48–52 Hz to avoid contamination through line noise. LFP data were transformed to the time-frequency domain using Morlet-wavelets with 8 cylces and a frequency resolution of 1 Hz. The transformed data were then normalized to the total power as a sum across the frequency ranges 3–47 Hz and 53–97 Hz per subject, and further on represented as % of the total sum. Power spectra were then averaged over 30 sec (30.4 ± 3.6 sec) during the resting state on each stimulation step and inspected visually for the presence of peaks in the beta frequency band. Furthermore, investigated mean frequency ranges for each stimulation step were theta (5–8 Hz), alpha (8–12 Hz), total beta (13–35 Hz), and low beta (13–20 Hz) and high beta (20–35 Hz) separately, as differential dynamics have been described previously. For assessment of post-DBS baseline recovery, low-beta-band activity (13–20 Hz) relative to the mean pre-DBS baseline was calculated for each second in the first 20 s after DBS cessation.

LFP and accelerometer traces were synchronized offline, accelerometer traces were cut to LFP recording duration and down-sampled to 250 Hz, then added as an additional channel to the LFP recording file.

Accelerometer traces were inspected visually, and movement times were defined manually in the z-axis of all accelerometer recordings. As different accelerometers and recording systems were used, accelerometer traces were smoothed with moving average kernel of 100 samples length, normalized over the entire recording time using z-scoring (X-mean/std) and averaged velocity per block and per stimulation step (2–4 blocks, mean 2.9 ± 0.4 blocks, smoothed at a smoothing kernel of 10^7^ samples). Subject 6 and subject 7 were tremor-dominant patients, subject 6 did not show clinically relevant bradykinesia and was excluded from the best clinical effect analysis.

Differences in power per frequency were tested using Monte Carlo paired permutation testing with a fixed number of 10.000 permutations. Standard deviation was calculated as the square root of the normalization of the sample by sample size-1. Velocity and low-beta values were tested for normal distribution and correlated per individual using Pearson/Spearman correlation.

A linear mixed effects model was fitted to assess the association between low-beta-band activity and change in velocity. ‘Subjects’ were included in the model as a ‘random effect’ to account for the hierarchical structure of the data arising from multiple testings per subject. Visual data inspection indicated an exponential relationship between low-beta frequency band activity and stimulation amplitude. Normalized frequency band activity was therefore logarithmized and a generalized linear mixed effects model was implemented. Models were compared using the Bayesian Information Criterion (BIC).

### Reporting summary

Further information on research design is available in the Nature Research Reporting Summary linked to this article.

## Supplementary information

Supplementary Figures

Reporting Summary

## Data Availability

The data and code that support the findings of this study are available from the corresponding author upon reasonable request.

## Code availability

All analysis was done in Matlab using the open-source Perceive toolbox (https://github.com/neuromodulation/perceive/) and the statistical parametric mapping toolbox (SPM12, UCL, London, UK). Additional code information is available from the corresponding author upon reasonable request.
