# Supplementary material for: Toward therapeutic electrophysiology: beta-band suppression as a biomarker in chronic local field potential recordings
Source: NPJ Parkinsons Dis. 2022 Apr 19;8:44. doi: 10.1038/s41531-022-00301-2 (PMC9018912; doi:10.1038/s41531-022-00301-2)
Supplement: Supplementary file 1 — Supplementary Figures [file 41531_2022_301_MOESM1_ESM.pdf]

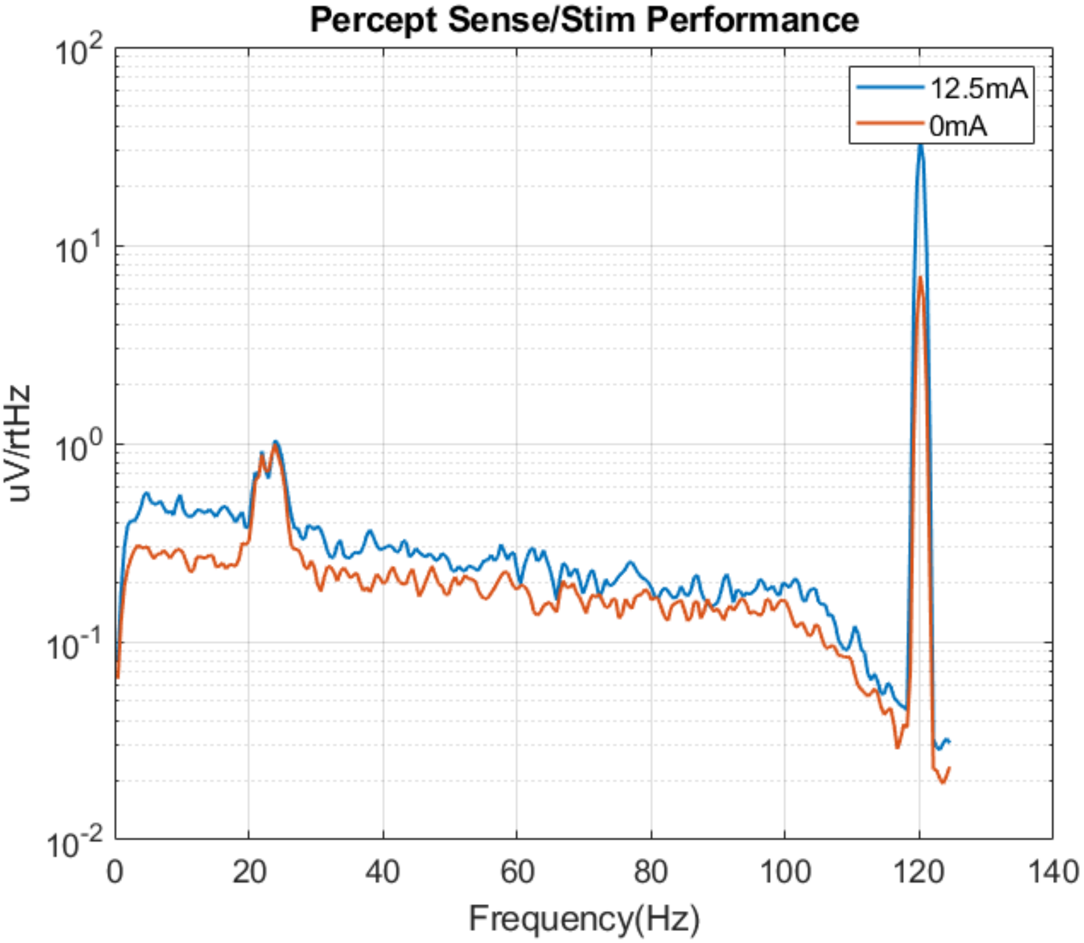

Supplementary Figure 1: Characterization of the Percept signal chain for stimulation artefact sensitivity

A test tone centered in the beta band region, with signal amplitude of 1uV/rtHz (approximately 2uVrms), was injected into the signal chain using a saline tank to mimic the conditions normally found in an implant. The measurement dipole used two electrodes symmetrically placed next to a stimulation electrode. The baseline (red) periodogram was recorded with 0mA applied to the stimulation electrode; note the residual artefact at 120Hz from the stimulation background circuits perturbing the signal chain. A 12.5mA, 90us, stimulation signal at 120Hz was then applied to the stimulation electrode centered between the measurement electrodes and returned to case. The impact of this large stimulation artefact was to elevate the noise floor of the sensing amplifier, but did not impact the signal level or frequency of the beta-band test tone. The impact of channel stimulation artefact is to elevate the measurement floor, but not change the measurement amplitude or frequency of physiological biomarkers.

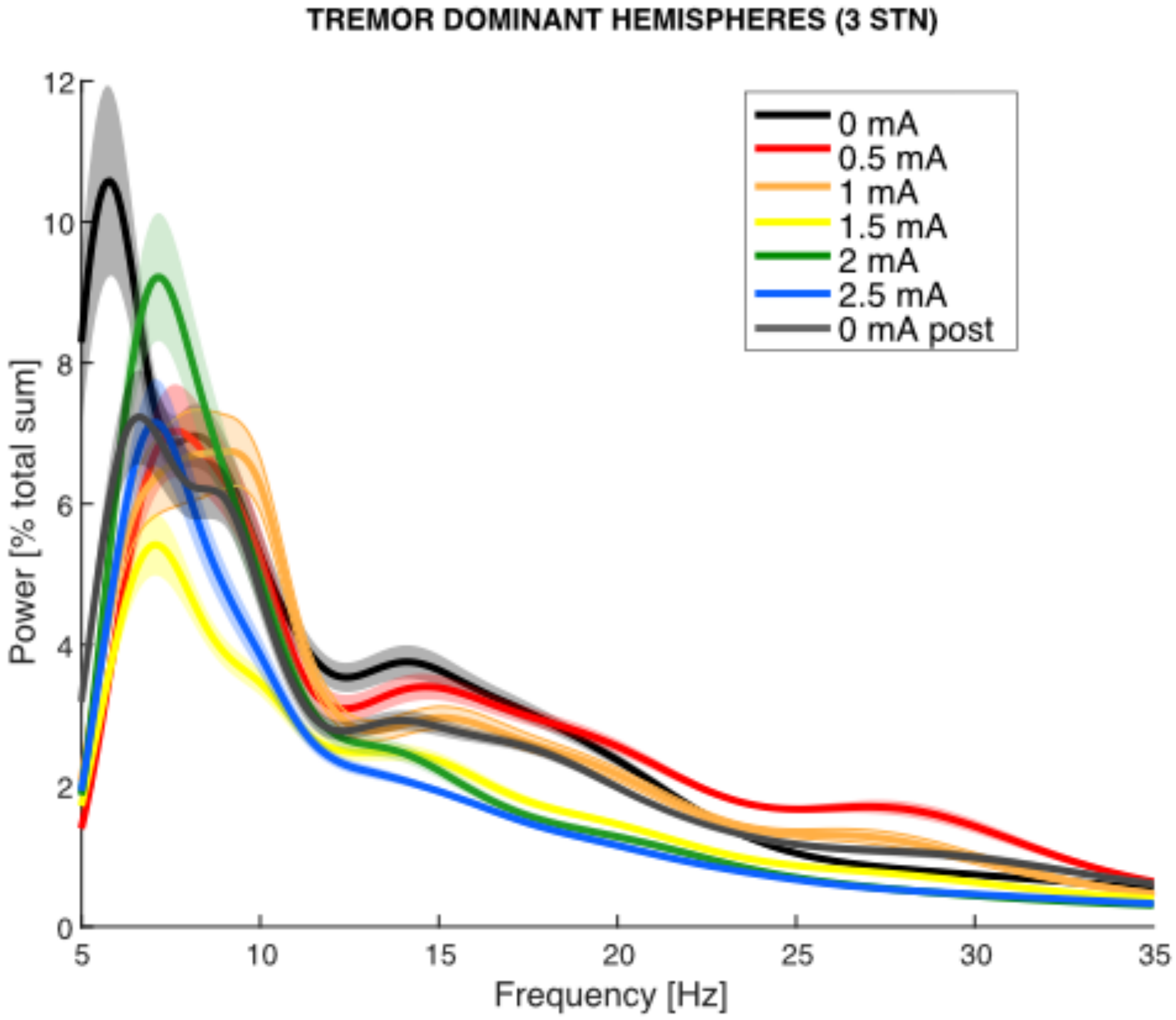

Supplementary Figure 2: Beta band suppression in a subgroup of tremor-dominant patients (3 STN)

Although there is a proportionally lower beta peak activity and higher low frequency activity in the tremor-dominant subgroup than in the bradykinetic patients, there is a consistent stepwise beta band suppression at clinically effective stimulation amplitudes.

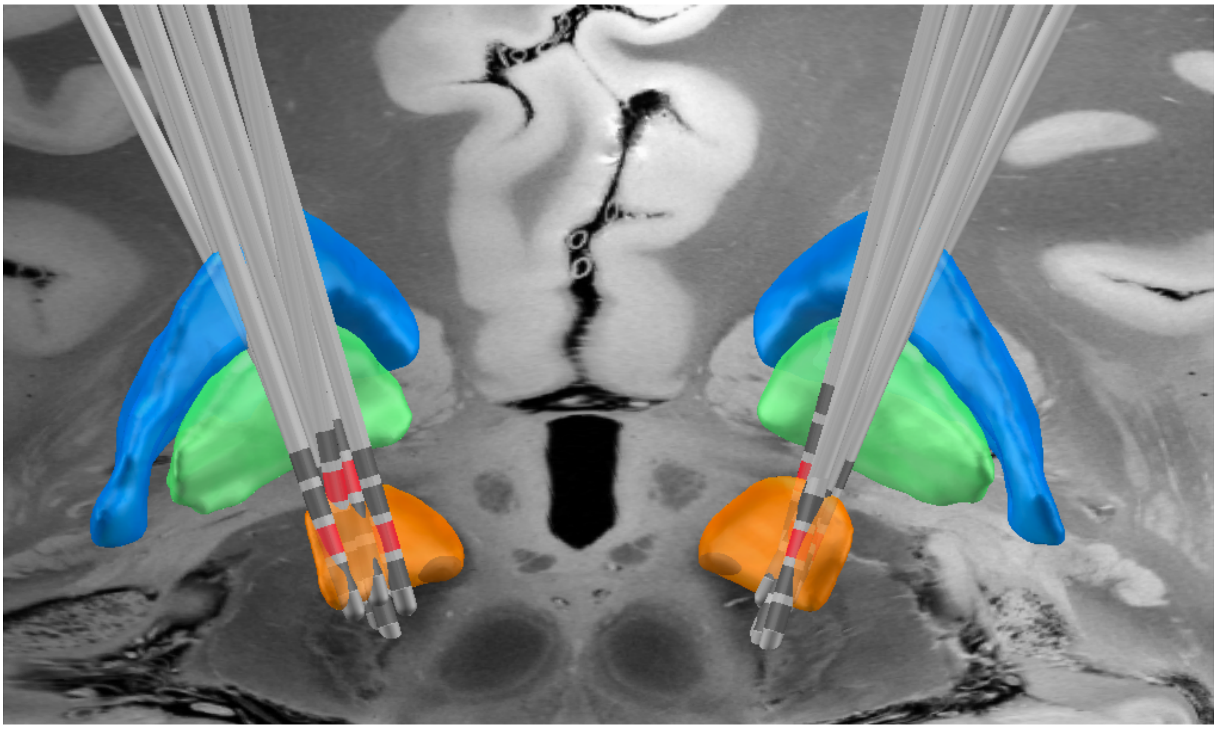

Supplementary Figure 3: DBS lead localization and active stimulation contacts
Reconstruction of localization of study cohort DBS-leads projected on the STN (orange). Active stimulation contacts for the monopolar review are marked in red color.
