# Supplementary material for: Toward therapeutic electrophysiology: beta-band suppression as a biomarker in chronic local field potential recordings
Source: NPJ Parkinsons Dis. 2022 Apr 19;8:44. doi: 10.1038/s41531-022-00301-2 (PMC9018912; doi:10.1038/s41531-022-00301-2)
Supplement: Supplementary file 2 — Reporting Summary [file 41531_2022_301_MOESM2_ESM.pdf]

nature portfolio

Corresponding author(s): Andrea A. Kühn
Movement Disorders and Neuromodulation Unit, Department of Neurology
Charité Universitätsmedizin, Charitéplatz 1, Berlin, 10117, Germany
Telelphone: 0049-(0)30-450660203
E-mail: andrea.kuehn@charite.de

Last updated by author(s): 07.01.2022

Reporting Summary

Nature Portfolio wishes to improve the reproducibility of the work that we publish. This form provides structure for consistency and transparency in reporting. For further information on Nature Portfolio policies, see our Editorial Policies and the Editorial Policy Checklist.

Statistics

For all statistical analyses, confirm that the following items are present in the figure legend, table legend, main text, or Methods section.

n/a	Confirmed	
☐	☒	The exact sample size (*n*) for each experimental group/condition, given as a discrete number and unit of measurement
☐	☒	A statement on whether measurements were taken from distinct samples or whether the same sample was measured repeatedly
☐	☒	The statistical test(s) used AND whether they are one- or two-sided<br>*Only common tests should be described solely by name; describe more complex techniques in the Methods section.*
☐	☒	A description of all covariates tested
☐	☒	A description of any assumptions or corrections, such as tests of normality and adjustment for multiple comparisons
☐	☒	A full description of the statistical parameters including central tendency (e.g. means) or other basic estimates (e.g. regression coefficient) AND variation (e.g. standard deviation) or associated estimates of uncertainty (e.g. confidence intervals)
☒	☐	For null hypothesis testing, the test statistic (e.g. *F*, *t*, *r*) with confidence intervals, effect sizes, degrees of freedom and *P* value noted<br>*Give P values as exact values whenever suitable.*
☐	☒	For Bayesian analysis, information on the choice of priors and Markov chain Monte Carlo settings
☒	☐	For hierarchical and complex designs, identification of the appropriate level for tests and full reporting of outcomes
☐	☒	Estimates of effect sizes (e.g. Cohen's *d*, Pearson's *r*), indicating how they were calculated

Our web collection on statistics for biologists contains articles on many of the points above.

Software and code

Policy information about availability of computer code

Data collection	All data was obtained using the Percept TM IPG (Medtronic), the motor task performance was obtained using 3-D accelerometers (TMSi, The Netherlands; Alpha Omega, Nazareth, Israel; Analog Devices, Norwood, USA).
Data analysis	All data were analyzed offline in MATLAB, using open source Perceive toolbox (https://github.com/neuromodulation/perceive/) and the statistical parametric mapping toolbox (SPM12, UCL, London, UK).

For manuscripts utilizing custom algorithms or software that are central to the research but not yet described in published literature, software must be made available to editors and reviewers. We strongly encourage code deposition in a community repository (e.g. GitHub). See the Nature Portfolio guidelines for submitting code & software for further information.

Data

Policy information about availability of data

All manuscripts must include a data availability statement. This statement should provide the following information, where applicable:

- Accession codes, unique identifiers, or web links for publicly available datasets
- A description of any restrictions on data availability
- For clinical datasets or third party data, please ensure that the statement adheres to our policy

The data and code that support the findings of this study are available from the corresponding author upon reasonable request.

Field-specific reporting

Please select the one below that is the best fit for your research. If you are not sure, read the appropriate sections before making your selection.

☒ Life sciences ☐ Behavioural & social sciences ☐ Ecological, evolutionary & environmental sciences

For a reference copy of the document with all sections, see nature.com/documents/nr-reporting-summary-flat.pdf

Life sciences study design

All studies must disclose on these points even when the disclosure is negative.

Sample size	10 Parkinson's disease patients implanted with the novel Percept IPG were included in the study. Similar sample sizes have previously been published for similiar research questions.
Data exclusions	Data was inspected visually and recordings with ECG contamination were identified in relation of the delta oscillatory activity and beta band activity, subjects with strong stimulation aliasing were excluded (see Table 1).
Replication	not applicable
Randomization	not applicable
Blinding	not applicable

Reporting for specific materials, systems and methods

We require information from authors about some types of materials, experimental systems and methods used in many studies. Here, indicate whether each material, system or method listed is relevant to your study. If you are not sure if a list item applies to your research, read the appropriate section before selecting a response.

Materials & experimental systems

n/a	Involved in the study	
☒	☐	Antibodies
☒	☐	Eukaryotic cell lines
☒	☐	Palaeontology and archaeology
☒	☐	Animals and other organisms
☐	☒	Human research participants
☐	☒	Clinical data
☒	☐	Dual use research of concern

Methods

n/a	Involved in the study	
☒	☐	ChIP-seq
☒	☐	Flow cytometry
☒	☐	MRI-based neuroimaging

Human research participants

Policy information about studies involving human research participants

Population characteristics	10 Parkinson's disease patients (6 females/4 males) from 3 DBS centers, demographic information is provided in a table in the main manuscript.
Recruitment	Patients were recruited at the three month's follow-up visitation/outpatient clinic in the centers involved in the study. The opportunity to participate in the study was offered to all patients with the Percept IPG at 3-months-follow-up, and during outpatient clinic visits.
Ethics oversight	The study was approved by the local ethics committees of the Charité Universitätsmedizin Berlin (EA2/256/60) and the Medical Faculty of Heinrich-Heine-University Düsseldorf (Study No. 2019-629_2), and was conducted in accordance with the ethical standards set by the Declaration of Helsinki.

Note that full information on the approval of the study protocol must also be provided in the manuscript.

Clinical data

Policy information about clinical studies

All manuscripts should comply with the ICMJE guidelines for publication of clinical research and a completed CONSORT checklist must be included with all submissions.

Clinical trial registration	not a clinical trial
Study protocol	All recordings were performed separately for each hemisphere after withdrawal of dopaminergic medication for at least 12 hours

Study protocol	(OFF medication state). Recordings OFF-stimulation were performed after a washout-phase of at least 30min.<br>Initially, we evaluated the artefact status and the power spectra generated for all possible recording configurations in the BrainSense Survey/BrainSense Signal Check modes using the Medtronic clinician programmer. During chronic sensing only the middle two contacts can be used for stimulation. From the contact pairs adjacent to the two possible stimulation contacts (right: 1+2/left: 9+10), we selected the bipolar contact pair with the highest beta peak for the recordings (right: 0-2, 1-3; left: 8-10, 9-11).<br>During the monopolar review, patients were seated comfortably in an arm chair. Following a rest recording of 60 sec, patients conducted 2-3 blocks of 10 sec finger tapping (MDS Unified Parkinson's Disease Rating Scale (UPDRS)-III item 3.7/3.8) of both hands, with 10 sec rest between each block. For each hemisphere in separate recordings, the stimulation was unilaterally increased in steps of 0.5 mA (Fig. 1) up to the presentation of side-effects. On each stimulation level, the rest and finger tapping assessments, as described above, were conducted.
Data collection	Local field potentials (LFP) were recorded using the Percept IPG, data were sampled at 250 Hz, streamed to the Medtronic clinician programmer, exported to the json-file format and saved to a personal computer. The motor task performance was objectified using 3-D accelerometers (n=7: TMSi, The Netherlands; n=1: Alpha Omega, Nazareth, Israel; n=2: Analog Devices, Norwood, USA). Additionally, the improvement of overall motor performance during stimulation was assessed using MDS UDPRS Part-III scores OFF medication.<br>Clinical data was retrieved from our database during the recording/main analysis period (June 2020-August 2021) and during the revision period (December 2021-January 2022).
Outcomes	Modulation of electrophysiological data through deep brain stimulation, especially in the beta frequency band, and motor improvement as velocity recorded with the accelerometer recordings were the outcome measures in the main analysis.
